# Adult Palatum as a Novel Source of Neural Crest-Related Stem Cells

**DOI:** 10.1002/stem.104

**Published:** 2009-08

**Authors:** Darius Widera, Christin Zander, Meike Heidbreder, Yvonne Kasperek, Thomas Noll, Oliver Seitz, Belma Saldamli, Holger Sudhoff, Robert Sader, Christian Kaltschmidt, Barbara Kaltschmidt

**Affiliations:** a Institute of Cell Biology, Faculty of Biology, University of BielefeldBielefeld, Germany; b Molecular Neurobiology Group, Faculty of Biology, University of BielefeldBielefeld, Germany; c Institute of Cell Culture Technology, University of BielefeldBielefeld, Germany; d Klinik für Mund-, Kiefer- und Plastische GesichtschirurgieFrankfurt, Germany; e Klinik für Hals-, Nasen- und Ohrenheilkunde, Kopf- und Halschirurgie, Staedtische Kliniken BielefeldBielefeld, Germany

**Keywords:** Neural crest, Sox2, Klf4, Oct4, c-Myc, Novel human stem cell source, Tissue stem cells

## Abstract

Somatic neural and neural crest stem cells are promising sources for cellular therapy of several neurodegenerative diseases. However, because of practical considerations such as inadequate accessibility of the source material, the application of neural crest stem cells is strictly limited. The secondary palate is a highly regenerative and heavily innervated tissue, which develops embryonically under direct contribution of neural crest cells. Here, we describe for the first time the presence of nestin-positive neural crest-related stem cells within Meissner corpuscles and Merkel cell-neurite complexes located in the hard palate of adult Wistar rats. After isolation, palatal neural crest-related stem cells (pNC-SCs) were cultivated in the presence of epidermal growth factor and fibroblast growth factor under serum-free conditions, resulting in large amounts of neurospheres. We used immunocytochemical techniques and reverse transcriptase-polymerase chain reaction to assess the expression profile of pNC-SCs. In addition to the expression of neural crest stem cell markers such as Nestin, Sox2, and p75, we detected the expression of Klf4, Oct4, and c-Myc. pNC-SCs differentiated efficiently into neuronal and glial cells. Finally, we investigated the potential expression of stemness markers within the human palate. We identified expression of stem cell markers nestin and CD133 and the transcription factors needed for reprogramming of somatic cells into pluripotent cells: Sox2, Oct4, Klf4, and c-Myc. These data show that cells isolated from palatal rugae form neurospheres, are highly plastic, and express neural crest stem cell markers. In addition, pNC-SCs may have the ability to differentiate into functional neurons and glial cells, serving as a starting point for therapeutic studies. Stem Cells *2009;27:1899–1910*

## INTRODUCTION

The neural crest was first described in the development of the chick embryo as the *Zwischenstrang*, the intermediate cord, as it appeared between the neural cord and the future ectoderm [1].

During vertebrate embryo development, neurulation results in the neural tube, forming above the notochord by cell movement of the neural plate. This invagination later builds the brain and the spinal cord and is covered by the epidermis after the completion of the process.

Neural crest cells have since been shown to originate at the fusion line, from where they migrate after neurulation to give rise to various populations of cells in the adult body. They also have the ability for self-renewal. Thus, neural crest cells can be defined as a stem cell population [2].

By migration from the neural cord, they give rise to many cell types, including cartilage, pigment cells, and neurons of both the peripheral and the autonomic nervous system.

Two main migratory pathways of neural crest cells can be observed: the dorsolateral and ventral paths, which result in different types of tissue. In their target tissues, the uncommitted neural crest cells differentiate into cells of both mesodermal and ectodermal type, giving the neural crest its description as a probable fourth germ layer [3,4]. Growth factors and the specific microenvironment in target tissues seem to be important for neural crest differentiation, as neural crest cells have been shown to change their properties when transplanted into different tissues during development [5–7].

Neural crest cells could be termed a dormant stem cell population in the adult, as they are pluripotent at first, but with migration they become restricted during development. Neural crest cells that form the vertebrate head skeleton migrate and interact with surrounding tissues to shape the skull. Interestingly, defects in these processes underlie several human craniofacial syndromes [8–10].

Palate is a highly regenerative and richly innervated craniofacial tissue. It is well described that wounds within oral mucosa heal rapidly [11–13]. This capability for rapid regeneration may be explained either by the presence of growth factors, such as basic fibroblast growth factor in the saliva, or by the potential presence of at least one stem cell type within the tissue.

Several recent studies showed that neural crest-related stem cells can be isolated from mammalian craniofacial tissues such as periodontal ligament [14,15] or dental pulp [16]. These cells have in common that they form neurosphere-like clusters and proliferate in serum-free culture in the presence of fibroblast growth factor-2 (FGF-2) and epidermal growth factor (EGF).

In this study we identified cells positive for the neural stem cell/neural crest-specific intermediate filament nestin adjacent to Meissner corpuscles and Merkel cell-neurite complexes within palatal ridges (*palatal rugae/rugae palatinae*).

These cells were characterized by expression of nestin, the transcription factor Sox2, and additional neural crest markers such as Slug or Snail.

In addition to the expression profile, the cellular properties of rat palatal neural crest-related stem cells (pNC-SCs) were determined. Finally, we investigated the potential presence of palatal stem cell pools within the human palate.

## MATERIALS AND METHODS

### Preparation of Cryosections

Wistar rats were decapitated when older than 23 days (adult). Briefly, the whole mucoperiosteum was removed using a dental scraper, embedded in optimal cutting temperature compound (Miles-Bayer, Leverkusen, Germany, http://www.bayer.de) and quick-frozen in −40°C cold isopentane. Cryostat sections (10 μm) were cut and mounted on polylysine-coated glass slides (Menzel-Gläser, Braunschweig, Germany, http://www.menzel.de). Palate slides were fixed in −20°C cold methanol for 5 minutes and processed for immunocytochemistry as stated below.

### Hematoxylin and Eosin Staining of Palatum Sections

Palatal cryostat sections were prepared as described above. After fixation with methanol at −20°C for 5 minutes, the sections were incubated in absolute ethanol for 2 minutes followed by incubation in 95% ethanol for 2 minutes and in 70% ethanol for 2 minutes. Subsequently, sections were briefly washed in distilled water and stained in Harris/Mayer hematoxylin solution (1:1; Roth, Karlsruhe, Germany, http://www.carl-roth.de/). After differentiation in 1% acid alcohol for 30 seconds, the sections were washed with H_2_O for 1 minute, followed by counterstaining with eosin-phloxine B solution (Roth) for 1 minute. After dehydration through 95% alcohol and incubation in xylene for 2 × 5 minutes, sections were mounted with Entellan (Merck, Darmstadt, Germany, http://www.merck.com). The sections were examined using Keyence BZ 8000, a phase-contrast microscope (Keyence, Neu-Isenburg, Germany, http://www.keyence.de).

### Cell Isolation and Culture of pNC-SCs

All palatal tissue was extracted from adult Wistar rats according to local guidelines (Bezirksregierung Duesseldorf). Namely, rats were killed by decapitation followed by the removal of the whole mucoperiosteum using a dental scraper. The tissue of 3–5 Wistar rats was chopped using a McIlwain tissue chopper and collected in ice-cold Hank's Buffered Salt Solution (HBSS)-glucose solution (HBSS from Gibco, Eggenstein, Germany, http://www.invitrogen.com) containing 300 mg/ml d-glucose (Sigma, Deisenhofen, Germany, http://www.sigmaaldrich.com) followed by digestion with 1.33 mg/ml trypsin (Sigma), 0.7 mg/ml hyaluronidase (Sigma), 200 U/ml DNAse (Sigma), and 0.2 mg/ml kynurenic acid (Sigma) for 30 minutes at 37°C. The tissue was then passed through a 100-μm cell strainer (BD Falcon, Heidelberg, Germany, http://www.bdbiosciences.com) and transferred to ice-cold Earle's Balanced Salt Solutions containing 15 mM HEPES and 0.04 g/ml bovine serum albumin.

pNC-SCs were cultured in serum-free media (Dulbeccos modified Eagles medium [DMEM]/F12; Gibco, Karlsruhe, Germany, http://www.gibco.invitrogen.com) containing basic FGF-2 (20 ng/ml; Chemicon, Hofheim, Germany, http://www.chemicon.com), EGF (20 ng/ml; R&D Systems, Wiesbaden, Germany, http://www.rndsystems.com), and B27 supplement (Gibco) as described earlier [17]. Primary neurospheres were dissociated at days 8–10 using Accutase (PAA Laboratories, Pasching, Austria, http://www.paa.at) and 100-μm cell strainer (BD Falcon) to derive secondary neurospheres. The subculturing protocol consisted of neurosphere passaging every 3–4 days with whole culture media change (and freshly added growth factors). In all assays neurospheres in passages 6–20 were used.

### Immunocytochemistry

Neurospheres were harvested on microscope slides by cytospin centrifugation (40*g*, 10 minutes; Thermo Shandon Inc., Dreieich, Germany, http://www.thermo.com). Fixation was performed in phosphate-buffered 4% paraformaldehyde (pH 7.4) (4% wt/vol paraformaldehyde, 100 mM NaH_2_PO_4_, and 0.4 mM CaCl_2_) for 60 minutes at 4°C followed by three washing steps in 1× PBS for 5 minutes. Blocking was done in 5% appropriate serum for 30 minutes at 23°C followed by incubation with primary antibodies for 1 hour at 23°C at the following dilutions: anti-nestin, 1:100 (Chemicon Milipore, Schwalbach, Germany, http://www.chemicon.com); Sox2, 1:150 (Sigma); rabbit anti-glial fibrillary acidic protein (anti-GFAP), 1:100 (Chemicon); mouse anti-β-III-tubulin, 1:100 (Promega, Madison, WI, http://www.promega.com); anti-microtubule-associated protein-2 (MAP2), 1:100 (Chemicon); anti-neurofilament-M, 1:100 (Chemicon); and mouse-anti-p65, 1:100 (Chemicon). The secondary fluorochrome-conjugated antibodies were diluted 1:300 (Alexa 555, 1 hour at 23°C; Molecular Probes Inc., Göttingen, Germany, http://probes.invitrogen.com). Nuclear counterstaining was performed with SYTOX green (1:20000; Molecular Probes) or antibody staining was visualized using confocal laser scanning microscopy (LSM 510; Carl Zeiss, Jena, Germany, http://www.zeiss.com) and analyzed using ZEN software (Carl Zeiss).

### Reverse Transcription-Polymerase Chain Reaction

Total RNA from rat palatum neural crest stem cells, liver, cerebellum, cortex, and heart and human tissue samples was isolated using the Quiagen RNeasy Mini Kit, which was used according to manufacturer's guidelines. cDNA was synthesized in a 20-μl reaction, containing 40 U of M-MuLV reverse transcriptase, 20 U of RiboLock Ribonuclease inhibitor, 0.2 μg of random hexamer primer, 5× reaction buffer, and deoxynucleosidetriphosphates (dNTP) mix. Polymerase chain reaction (PCR) was performed in a 25-μl reaction using the Finnzymes High-Fidelity Phusion-Taq Polymerase Kit, containing 0.02 U/μl of Phusion DNA polymerase, 1× Phusion High Fidelity buffer, 10 mM dNTPs (200 μM each), and 0.5 μM primers (Metabion, Martinsried, Germany, http://www.metabion.com). The cycling conditions comprised an initial denaturation of 30 seconds at 98°C and 38 cycles of 10 seconds at 98°C, 20 seconds at the appropriate temperature, and 20 seconds at 72°C followed by final elongation for 5 minutes at 72°C. The primer sequences are provided in Table 1.

**Table 1:** ABS

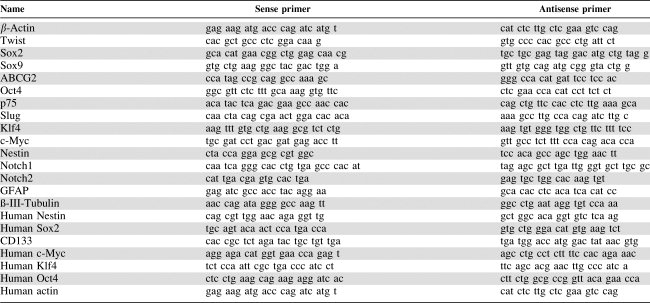

### Proliferation Assays

A number of 1.0 × 10^5^/ml dissociated nonadherent pNC-SCs were cultivated for a period of 4 days. Cell numbers per milliliter were determined every 24 hours by counting using a Neubauer improved hematocytometer (Sigma). Beforehand, cells were harvested by centrifugation in a Biofuge (Heraeus Sepatech, Osterode, Germany, http://www.thermo.com) at 212*g* for 5 minutes and chemically and mechanically dissociated and cell numbers were determined in triplicate.

Differences in proliferation between the different time points were assessed by two-way ANOVA followed by post hoc *t* test with Bonferroni correction using Graph Pad's Prism. *p* ≤ .05 was considered significant. Population doubling time was calculated using the algorithm provided by http://www.doubling-time.com.

### Limited Dilution Assay

To derive clonally related neurospheres, a limited dilution assay was used. After enzymatic and mechanical dissociation, the cells were diluted in cultivation media to obtain theoretically 1 cell per 100 μl and were subsequently placed into 96-well flat-bottom microtiter plates. In total, 384 wells were analyzed for the presence of single cells 4 hours after plating. At 96 hours the wells were examined for newly formed neurospheres using an inverse microscope equipped with phase contrast.

### Cell Cycle Analysis by DNA Content Using Fluorescence-Activated Cell Sorting

Nuclear DNA staining was done devoid of cytoplasmic membranes. Therefore, 5 × 10^5^ cells per sample were harvested and plasma membranes lysed for 30 minutes by a hypotonic lysis solution (0.1% wt/vol sodium citrate, 0.1% vol/vol Triton X-100). Concomitantly, nuclei were stained with 0.1% vol/vol propidium iodide (PI) and aggregates removed by filtration through a 40-μm mesh. PI staining was analyzed on a FACSCalibur (FACS, fluorescence-activated cell sorting) flow cytometer (Becton, Dickinson and Company, Germany, http://www.bd.com) and data subsequently processed with the CellQuest software (Becton, Dickinson and Company).

### Preparation of Rat Metaphase Chromosomes

A number of 1 × 10^6^ cells each were incubated for 2 hours at 37°C in a medium containing colcemide (0.05 μm/ml) to arrest cells in mitotic metaphase. After treatment with prewarmed hypotonic solution (0.075 M KCl) for 15 minutes at 37°C, the cells were progressively fixed three times in methanol/acetate (3:1). The suspension was dropped from a height of 100 cm onto microscope slides and air-dried at room temperature. Chromosomes were stained with DAPI (50 ng/ml) and mounted with Moviol (Hoechst, Frankfurt aM, Germany).

### Cell Differentiation

Neuronal differentiation was induced by pretreatment of 1.0 × 10^5^/ml suspension cells with 5 μM retinoic acid for 4 days in the presence of FGF-2 and EGF. Afterward, the cells were dissociated and plated on poly-l-lysine and laminin-coated glass coverslips in 12-well plates under withdrawal of growth factors. Cultures were grown for 4 days in a Neurobasal medium (Gibco-BRL, Gaithersburg, MD, http://www.gibcobrl.com) supplemented with B27 (Gibco), fixed with phosphate-buffered 4% paraformaldehyde (pH 7.4) for 1 hour at 4°C, and processed for immunocytochemistry described above.

For glial differentiation cell suspensions of pNC-SCs were cultured in DMEM/F12 (1:1) (Gibco) with 10% fetal calf serum (FCS; Gibco) and growth factor deprivation and plated on glass coverslips in 12-well plates at a density of 1 × 10^5^ cells. After 4 days of growth, cultures were fixed and stained.

### Isolation of Human Palatal Tissue

Oral mucosa samples from the hard palate (paracrestal location) or from the attached vestibular gingiva were obtained from donors during elective dentoalveolar surgery, after an informed consent. Wedge-shaped samples measuring approximately 5 mm × 3 mm were excised with a scalpel blade no. 15 from the margin of the surgery access flap. These were immediately transferred into sterile 15-ml tubes filled with Dulbeccos phosphate-buffered saline (PAA Laboratories) precooled to 4°C. The samples were transferred onto ice immediately in the laboratory. The supernatant was removed and the samples were pelleted at 4500 rcf, 4°C for 5 minutes (Labofuge 400R; Thermo Shandon Inc.). The remaining supernatant was carefully removed using micropipettes. The sealed tubes containing the pelleted tissue samples were immersed in liquid nitrogen for 5 minutes and then stored in a freezer at −80°C until analysis.

## RESULTS

### Localization of Nestin-Positive Cells Within the Palate of Adult Wistar Rats

**Figure 1:**
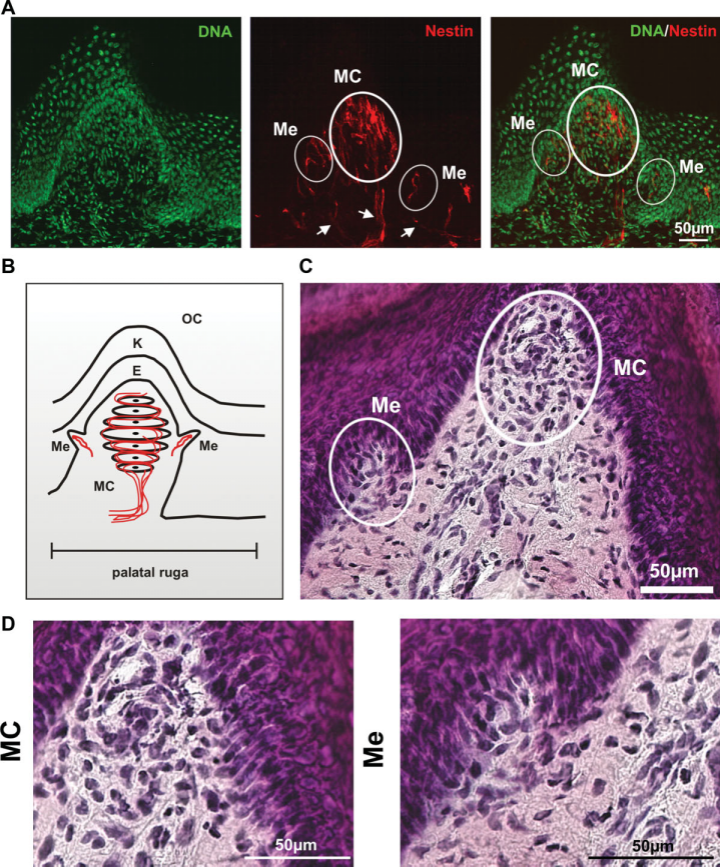
Nestin-positive progenitor cells within adult rat palate are localized adjacent to Meissner corpuscles and Merkel cell-neurite complexes.**A:** Saggital section along palatal ruga. Sections were immunostained against the stem cell-specific intermediate filament nestin and analyzed by confocal microscopy. Nestin-positive progenitor cells are localized in the mechanoreceptors MC and Me. A few nestin filaments are also present adjacent to innervation of MC and Me (marked by arrows). Bar = 50 μm. **B:** Schematic drawing of MC and Me within the submucosa of palatal ruga: (**E:** epidermis, **K:** keratinized *stratum corneum*). **C:** Hematoxylin and eosin (HE) staining of saggital sections along palatal ruga. Please note the typical MC and Me structures. Bar = 50 μm. **D:** High-magnification photograph of HE-stained Meissner corpuscles and Merkel cell-neurite complexes. Bar = 50 μm. Abbreviations: MC, Meissner corpuscles; Me, Merkel cell-neurite complexes; OC, oral cavity.

**Figure 2:**
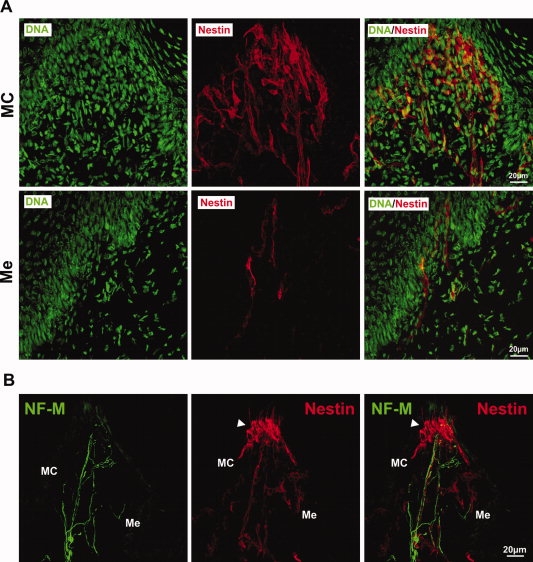
High-magnification confocal images of saggital sections along palatal rugae.**A:** Details from immunostained sections along palatal rugae show that Meissner corpuscles contain a higher amount of nestin-positive cells than Merkel cell-neurite complexes. Bar = 20 μm. **B:** Double staining of sections against NF-M and nestin. Nestin-positive cells within MC mainly are localized at the summit of the ruga, whereas the nestin filaments of Me are located in the deeper aspect of the rugal wall. The nerve fibers, visualized via NF-M staining, are partially co-localized with the nestin filaments in the central area of MC. At the summit of MC a high amount of nestin, but only few neurofilaments, could be detected. Bar = 20 μm. Abbreviations: MC, Meissner corpuscles; Me, Merkel cell-neurite complexes; NF-M, neurofilament-M.

To examine the predicted presence of neural crest stem cell-like cell types within the palate of adult Wistar rats, cryostat sections of the whole mucoperiosteum were prepared and immunostained for the stem cell-specific intermediate filament nestin. Here, we were able to detect highly innervated nestin-positive cells within palatal rugae adjacent to Meissner corpuscles (MC) and Merkel cell-neurite complexes (Me) (Figs. 1A, 2A). Hematoxylin and eosin staining revealed the typical structure of these mechanoreceptors (Fig. 1C). This is the first report on mammalian nestin-positive cell population adjacent to MC. Double staining of sections against neurofilament-M (NF-M) and nestin demonstrated that nestin-positive cells within MC mainly are localized at the summit of the ruga, whereas the nestin filaments of Me are located in the deeper aspect of the rugal wall (Fig. 2B). The nerve fibers, visualized via NF-M staining, are partially co-localized with the nestin filaments in the central area of MC. At the summit of MC a high amount of nestin, but only few neurofilaments, could be detected (Fig. 2B).

### Cell Isolation and Culture of pNC-SCs

**Figure 3:**
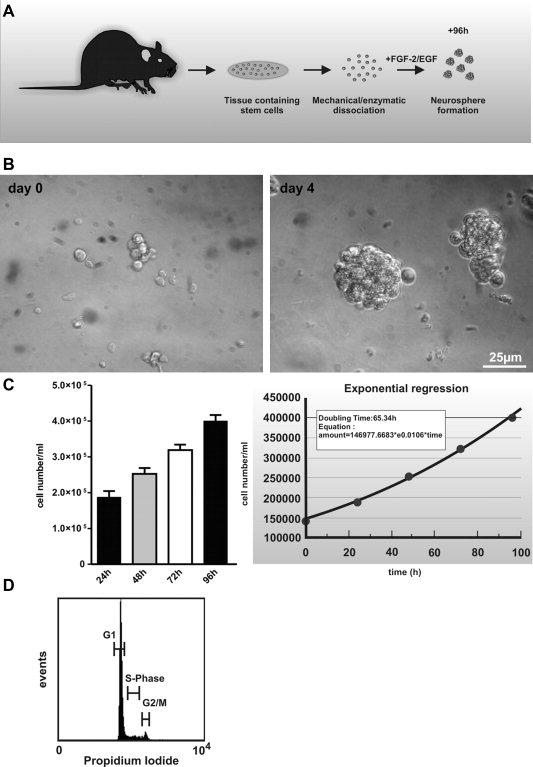
Isolated cells from adult palate form neurosphere-like clusters if cultivated under serum-free conditions in the presence of FGF-2 and EGF. **A:** Schematic diagram of the isolation protocol. After removal, the palatal tissue of adult rats was dissociated enzymatically and mechanically. The cells were cultured in the presence of FGF-2 and EGF. After at least 4 days of culture, freely floating, neurosphere-like aggregates were observed. **B:** Cultured palate-derived neural crest stem cells (pNC-SCs) form neurospheres, similarly to subventricular zone-derived neural stem cells, when cultured for 4 days in serum-free medium in the presence of FGF-2 and EGF. Bar = 25 μm. **C:** pNC-SCs are moderately proliferative. Proliferation of pNC-SCs was analyzed by counting the total cell number after dissociation of the spheres at different time points. pNC-SCs proliferate moderately in vitro with a population doubling time of 65.34 hours. **D:** pNC-SC cultures showed a PI fluorescence-activated cell sorting (FACS) pattern typical for a diploid culture. In vitro, proliferating cells often escape from growth regulation, resulting in ploidy changes. The ploidy was tested using FACS analysis of PI-stained DNA from cultivated, proliferating pNC-SCs. Abbreviations: EGF, epidermal growth factor; FGF-2, fibroblast growth factor-2; PI, propidium iodide.

Whole preparations of the mucoperiosteum were dissociated and cultivated under serum-free conditions in the presence of FGF-2 and EGF (Fig. 3A). All approaches were successfully propagated as neurosphere cultures after at least 4–6 days (Fig. 3B). Because of their putative origin, we called these cells palatal neural crest-related stem cells (pNC-SCs). Neurosphere cultures can be kept in culture for at least 20 passages without losing their chromosomal stability and capacities for proliferation and differentiation [18].

### pNC-SCs Are Proliferative and Form Secondary Neurospheres with the Frequency of 1.8%

Proliferation of pNC-SCs was analyzed by determining total cell numbers to avoid potential artifacts of 5-bromo-2-deoxyuridine incorporation such as labeling of DNA repair sites. The cell number was determined every 24 hours (Fig. 3C). Here, we showed that pNC-SCs proliferate without morphological signs of differentiation, indicating self-renewal of the cells. The population doubling time was determined as 65.34 hours (Fig. 3C). In an additional limited-dilution assay, we showed that pNC-SCs form secondary neurospheres with the frequency of 1.8% (data not shown).

### pNC-SCs Harbor Normal DNA Content and Chromosome Number

In vitro, proliferating cells often escape from growth regulation as a result of genetic abnormalities that manifest in alterations including ploidy and karyotype changes. In this approach ploidy was tested using FACS analysis of PI-stained DNA from proliferating pNC-SCs. Cells showed a propidium iodide FACS pattern typical for a diploid culture (Fig. 3D). Interestingly, no significant sub-G1-peak was detected. To confirm the genetic stability of cultivated pNC-SCs cultures, metaphase chromosomes were prepared and the chromosome number was determined. After 21 passages, pNC-SCs contained 42 chromosomes typical for a Wistar rat (data not shown).

### Characterization of pNC-SCs

**Figure 4:**
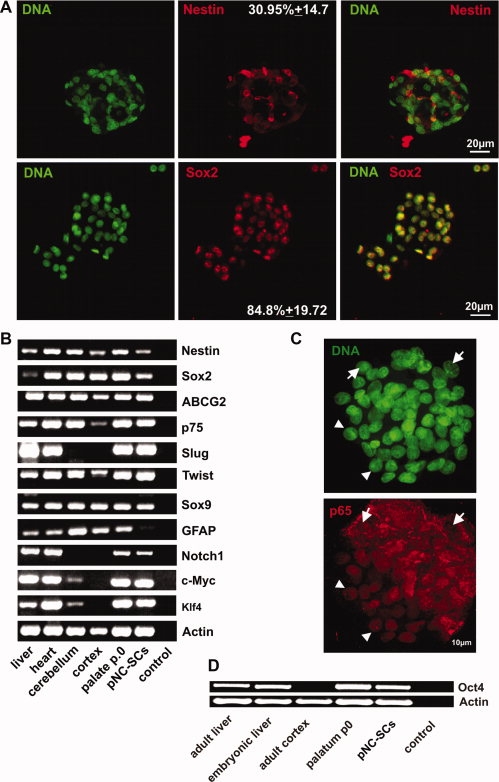
Analysis of the expression pattern of cultured pNC-SCs. **A:** pNC-SCs expressed the intermediate filament nestin and the transcription factor Sox2. Cytospin preparations of pNC-SCs were fixed and stained for nestin and Sox2. Approximately 30% of the cultivated cells expressed nestin, whereas ∼85% of the cells were positive for Sox2. **B:** RT-PCR (reverse transcriptase-polymerase chain reaction) verified the expression of the stem cell markers nestin and Sox2. pNC-SCs expressed the neural crest-specific transcripts p75, Slug, Sox9, Twist, and ABCG2. In addition, pNC-SCs were positive for the transcription factors c-Myc and Klf4. cDNA was normalized using the β-actin housekeeping gene. **C:** Distribution of nuclear factor-kappaB (NF-κB) in pNC-SCs. pNC-SCs were harvested by cytospin centrifugation, fixed, stained, and analyzed for subcellular NF-κB p65 distribution. The cells within the spheres showed heterogeneous distribution pattern. Approximately 62% of the cells showed nuclear p65 (marked by arrowheads), whereas ∼38% cells with cytoplasmic distribution were detected (arrows). Bar = 10 μm. **D:** RT-PCR analysis showed high expression of Oct4 in adult palate (palatum p0) and isolated pNC-SCs cultivated for 14 passages. Abbreviation: pNC-SCs, palate-derived neural crest stem cells.

In the newly propagated population of pNC-SCs, the protein expression of neural stem cell-specific biomarkers nestin and Sox2 was assayed using antibodies (Fig. 4A): 30.95% ± 14.7% of the pNC-SCs were anti-nestin immunoreactive, a characteristic of neural/neural crest stem cells; 84.8% of the cells also expressed the neural/neural crest stem cell-specific transcription factor Sox2. There was no expression of the neuronal differentiation marker β-III-tubulin (data not shown). In contrast, few cells showed an expression of GFAP (glial cell marker, data not shown).

Additionally, we studied the RNA expression of several markers using reverse transcriptase-polymerase chain reaction (RT-PCR) (Fig. 4B). The stem cell markers nestin and Sox2 were strongly expressed in all tested samples, indicating the presence of putative stem cells in all examined tissues and organs. RT-PCR verified the expression of GFAP. Demonstrably, pNC-SCs showed high expression of neural crest markers as p75, ABCG2, Slug, Twist, and Sox9. Interestingly, the RNA for Slug was absent in all CNS-related control samples (cortex and cerebellum). In addition, we could show a weak expression of Klf4 and c-Myc in the adult cerebellum but not in the adult cortex. As shown by in situ hybridizations for Klf4, this gene is transcribed in mouse adult cerebellar granule cells and not in cortex, whereas c-Myc transcription is restricted to Purkinje cells (supporting information Figs. 1, 2). In summary, our PCR results match the data gained by in situ hybridizations against Klf4 and c-Myc shown in the Allen Brain Atlas (http://www.brain-map.org [19]). Furthermore, pNC-SCs were positive for Notch1. Notch signaling has been described as crucial for cranial neural crest development and homeostasis [20,21]. Since Sox2, one of the so-called “magic four” [22], was highly expressed in pNC-SCs, we tested the potential expression of the other gene products Klf4, Oct4, and c-Myc as well. Here, we showed that pNC-SCs were positive not only for Sox2 but also for c-Myc and Klf4. Demonstrably, both palatal tissue and cultivated pNC-SCs expressed Oct4 (Fig. 4D). Oct4 has been recently shown to be solely sufficient for reprogramming of adult neural stem cells [23].

In addition, pNC-SCs expressed heterogeneously distributed nuclear factor-kappaB (NF-κB) p65 (Fig. 4C). Active NF-κB has been identified as one of the crucial transcription factors for the proliferation of neural stem cells [24,25]. Besides cells with cytoplasmic NF-κB (61.93%), several cells with nuclear NF-κB (38.05%) could be detected (Fig. 4C).

### pNC-SCs Can Be Differentiated Efficiently into Cells with Neuronal and Glial Phenotypes

**Figure 5:**
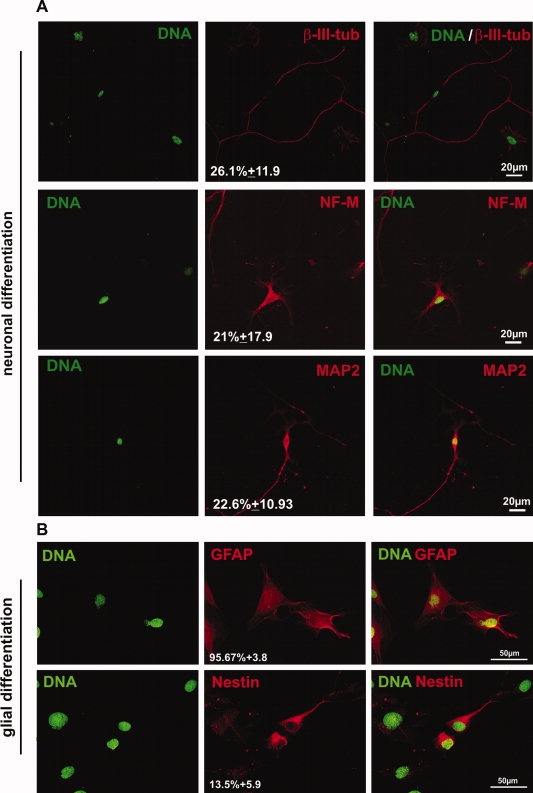
Differentiation potential of pNC-SCs. **A:** Palate-derived neural crest stem cells (pNC-SCs) differentiate into neuronal lineage if cultured in the presence of retinoic acid followed by plating on poly-d-lysine/laminin in the absence of growth factors. Cells were cultured in fibroblast growth factor-2/epidermal growth factor/retinoic acid-containing media for 4 days, followed by removal of the cytokines and plating on poly-d-lysine/laminin-coated culture chambers. Please note the expression of β-III-tubulin, NF-M, and MAP2. Bar = 20 μm. **B:** pNC-SCs can be differentiated into glial lineage when cultured for 4 days in the absence of growth factors and the presence of 10% fetal bovine serum. Please note the glial morphology and the robust expression of GFAP by nearly 96% of the cells. Approximately 13% of the pNC-SCs remained nestin-positive. Bar = 50 μm. Abbreviations: GFAP, glial fibrillary acidic protein; MAP2, microtubule-associated protein-2; NF-M, neurofilament-M.

To investigate the differentiation potential of pNC-SCs, the cultures were initially cultivated as aggregates in the presence of growth factors (FGF-2 and EGF) and 5 μM retinoic acid (RA) for 4 days. After at least 96 hours, the spheres were dissociated and single cells were plated on poly-d-lysine/laminin-coated coverslips in the absence of growth factors. One day after plating, a neuron-like morphology (bipolar cell bodies with elaborated processes) was already detectable (not shown). Four days after plating on poly-d-lysine/laminin-coated coverslips, the cells were fixed and processed for immunocytochemistry. Here, we detected high frequency of cells positive for neuronal markers (Fig. 5A). A percentage of the cells (26.1% ± 11.9%) expressed the early neuronal marker β-III-tubulin (TuJ). Furthermore, 21% ± 17.9% of differentiated pNC-SCs were immunopositive for the neuron-specific intermediate filament neurofilament-M (NF-M). A percentage of the cells (22.6% ± 10.93%) showed high expression of MAP2 (Fig. 5A).

To determine the potential for glial differentiation, the spheres were dissociated and cultured for 4 days in the presence of 10% FCS, after which a typical glial morphology was observed. Immunohistochemical analysis verified the glial lineage of pNC-SCs, as shown by the high expression of GFAP (Fig. 5B).

Taken together, the differentiation capacity of pNC-SCs provide further evidence for the stemness of this cell population.

### Identification of Human Palatal Cells Expressing Stem Cell Markers and the Transcription Factors Necessary for Reprogramming

**Figure 6:**
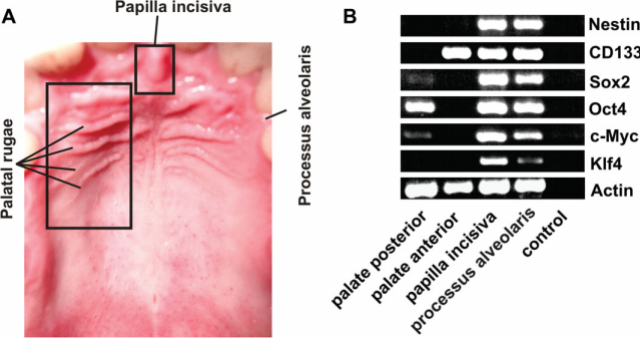
Human palatal tissue contains palate-derived neural crest stem cells.**A:** Overview photograph of human palatal structures. **B:** After tissue isolation and reverse transcription of the RNA, the expression pattern of various palatal regions was investigated by polymerase chain reaction. Besides anterior and posterior samples from the palate, samples from the papilla incisiva and the distal processus alveolaris maxillae were investigated. The highest expression of the human stem cell markers nestin and CD133 was detected within the papilla incisiva and the processus alveolaris. In addition, the tissue from this region showed high expression of all the so-called “magic four”: Sox2, c-Myc, Klf4, and Oct4. cDNA was normalized using the β-actin housekeeping gene.

To identify the potential stem cell pools within the human palate, we investigated anterior and posterior samples from hard palate. Additionally, samples from the *papilla incisiva* and the distal *processus alveolaris maxillae* were investigated (Fig. 6A). Using RT-PCR, we demonstrated the highest expression of the human stem cell markers CD133 and nestin within papilla incisiva and processus alveolaris (Fig. 6B). In addition, this population expressed high levels of Sox2, Klf4, Oct3/4, and c-Myc (Fig. 6B).

## DISCUSSION

In this study we describe for the first time a procedure for isolating neural crest-related stem cells from palatal rugae of adult rats.

In the head and the neck region of mammals, the neural crest yields not only ectodermal cell types such as neurons and glial cells but also mesodermal cells forming craniofacial cartilages, bones, and adipose tissue [26,27,3].

Thus, all structures of mammalian soft and hard palate are directly related to neural crest and neural crest stem cells. Demonstrably, cleft lip and palate may be caused by partial deficiency of mesencephalic neural crest cells [8]. Alternatively, cleft lip and palate may be caused by local changes in growth factors like transforming growth factor-β, bone morphogenetic proteins, and activins or by changes in the extracellular matrix and adhesion molecules (reviewed in [28]).

Several recent reports provided strong evidence for the persistence of neural crest stem cells in the adult [29,30,16,31,32,15,14,33,34]. These data suggest that neural crest stem cells could exhibit a dormant stem cell population in the adult, as they are pluripotent at first, but by migration become restricted during development. The persistence of these cells could be an explanation for the vast regenerative potential that several mammalian tissues show. In this context it is noteworthy that a palate is a highly regenerative and richly innervated cellular compound [11–13]. Thus, we hypothesized that the palate may contain dormant neural crest-related stem cells. In accordance with this prediction, we identified regions adjacent to Meissner corpuscles and Merkel cell-neurite complexes as the endogenous niche of the nestin-positive neural crest-related stem cells (Figs. 1, 2). Interestingly, Szeder et al. identified the neural crest as the origin of mammalian Merkel cells [35].

We were able to successfully isolate nestin- and Sox2-positive neural crest-related stem cells (Figs. 3B, 4A). The cells performed self-renewal if cultivated under serum-free conditions in the presence of FGF-2 and EGF (Fig. 3C). Limited dilution assays demonstrated secondary sphere-forming frequency of 1.8% (data not shown). In contrast to rat periodontal ligament-derived spheres having a sphere-forming frequency of 0.01% [15], this result is similar to the sphere-forming frequency of 1.5% showed by human corneal stromal cells cultured under sphere-forming conditions [36]. Interestingly, highly enriched rodent skin precursors from vibrissal follical isolated by microdissection showed a sphere-forming frequency up to 24.4% [37]. Since the isolated palatal tissue is heterogeneous, the primary spheres contain stem cells, precursors, and differentiated cells. Thus, an initial enrichment of pNC-SCs using microdissection may lead to elevated yield of sphere-forming cells.

The expression of nestin by 25% of isolated cells in contrast to the ability to form secondary spheres may be explained by the heterogeneity of the spheres and by the fact that nestin expression alone is not sufficient to ensure self-renewal.

It is well known that neural crest stem cells, like central nervous system progenitors, require FGF-2 to remain undifferentiated (reviewed in [34]). Administration of FGF-2 improves wound healing and suppresses scar formation after partial denudation of rat palate [38]. Spyrou and Naylor proposed an inhibited phenotypic change of granulation-tissue fibroblasts into myofibroblasts as the mechanism leading to improvement of wound healing [39]. Furthermore, FGF-2 has been shown to increase the angiogenesis in a rat wound-healing model [40]. Potentially enhanced proliferation of endogenous neural crest stem cells may be an additional mechanism explaining the phenomenon of improved wound healing.

Further analysis of the isolated and cultivated cells revealed the expression of several neural crest-specific markers such as Sox9, Twist, and Slug of p75 (Fig. 4B). Sox9 is mainly expressed during embryonic development, most notably in pre-chondrocytic and chondrocytic and pancreatic cells. It is important for neural crest formation, as it is responsible for epithelial-mesenchymal transition of neural crest cells during neurulation. In the adult, it maintains progenitor pluripotency and is important for proliferation and differentiation [41]. Absence of Sox9 in the developing brain leads to severe brain abnormalities [42]. Twist transcription factor is part of the basic helix-loop-helix (bHLH) family of transcription factors, including a zinc twist as DNA-binding zinc-protein. Twist seems to play a major role in the development of the mesoderm and the nervous system, especially lineage determination and differentiation. Like other members of the bHLH family, it seems to be important for determination and differentiation. Additionally, high levels of Twist seem to precede malignant growths of tumor tissue in the brain, such as gliomas [43].

Slug is a member of the Snail family of zinc-finger transcription factors, which have important roles in embryonic development. Slug is involved in cell movement, neural cell fate, and epithelial-mesenchymal transitions, an important feature during neural tube formation that eventually creates the neural crest. Mutations in Slug have been associated with several neural tube defects, such as the Waardenburg syndrome [44]. It is expressed in adult tissues during carcinogenesis, but is also re-expressed in several adult, but nonpathological, tissues, where it might take part in cell migration [45].

It has been demonstrated that adult cells, such as skin fibroblast, can be reprogrammed into a pluripotent state using four defined transcription factors [46]. In this approach human fibroblast cells were infected with retroviral vectors expressing the transcription factors Oct4, Sox2, Klf4, and c-Myc followed by a selection under embryonic stem cell culture conditions. These induced pluripotent stem cells (iPS) could differentiate into cell types of the three germ layers in vitro and in teratomas. To date, generation of iPS by expression of “magic four” from several sources including fibroblasts, lymphocytes, liver, stomach, and beta cells has been demonstrated [46–49].

Since exogenously induced or elevated expression of c-Myc and other transcription factors may cause tumorigenicity and retroviruses themselves can cause insertional mutagenesis, the reprogramming of somatic cells into iPS with a minimal number of factors is crucial for future clinical application. Recently, it has been clearly shown that progenitor cells expressing endogenously high levels of c-Myc and Sox2 or Sox2 alone can be efficiently reprogrammed using only three or two of the pluripotency related transcription factors [50,51]. In addition, Kim et al. quite recently showed that the viral expression of exogenous Oct4 in adult neural stem cells is sufficient to directly reprogram neural stem cells expressing endogenously Sox2, c-Myc, and Klf4 into pluripotent stem cells [23].

In this study we demonstrated, for the first time, that pNC-SCs isolated from the palate of adult rats express all the transcription factors sufficient for reprogramming: c-Myc, Klf4, Sox2, and Oct4 (Fig. 4B, 4D).

The expression pattern of isolated and cultivated pNC-SCs overlapped with the profile of palatal tissue before cultivation (Fig. 4B, 4D). This may be explained by the presence of tissue-resident stem cells and precursors within the palate. The expression of Sox2 and Oct4 in pNC-SCs compared to that in palatal tissue was slightly downregulated. Since the expression of pluripotency genes and stemness-related genes is strictly regulated during the commitment of stem cells, this phenomenon can be interpreted by partial differentiation of cells within the spheres. Besides the expression of the neural crest and stemness markers by pNC-SCs, we detected nestin, Sox2, and the neural crest markers p75, Twist, and Sox9 in all tested tissue samples. This result may be explained by the presence of dormant tissue-resident neural crest stem cells in several organs such as the heart [32].

In addition, we identified a novel source of human stem cells within the adult palate that express endogenously all of the “magic four” (Fig. 6). This newly described human stem cell population within papilla incisiva might represent a promising source of cells for reliable reprogramming and therapeutical use.

Since aberrant DNA content is a widely observed phenomenon in cultivated cells, we examined the DNA content and chromosome number of pNC-SCs. Since the pNC-SCs have a normal DNA content (Fig. 3D) and chromosome number, the method used here is suitable for potential clinical approaches if adapted to human material.

Finally, we showed that pNC-SCs are able to differentiate into neuronal and glial lineage as well (Fig. 5). The plasticity is another important requirement in any neural crest stem cell-based clinical approach. In this context we demonstrated that RA treatment efficiently induced neuronal fate in pNC-SCs as shown by the high levels of neuron-specific markers such as β-III-tubulin, MAP2, and neurofilament (Fig. 5A). When they were cultured in the presence of 10% FCS, pNC-SCs differentiated into a glial lineage, as demonstrated by glial morphology and GFAP expression (Fig. 5B).

Taken together, our study suggests that neural crest stem cells derived from mammalian palate could be an alternative, easily accessible source of multipotent or, after reprogramming, pluripotent adult stem cells for clinical and research use.
