# Supplementary figures and images for: Adult Palatum as a Novel Source of Neural Crest-Related Stem Cells
Source: Stem Cells. 2009 Aug;27(8):1899–910. doi: 10.1002/stem.104 (PMC2798069; doi:10.1002/stem.104)

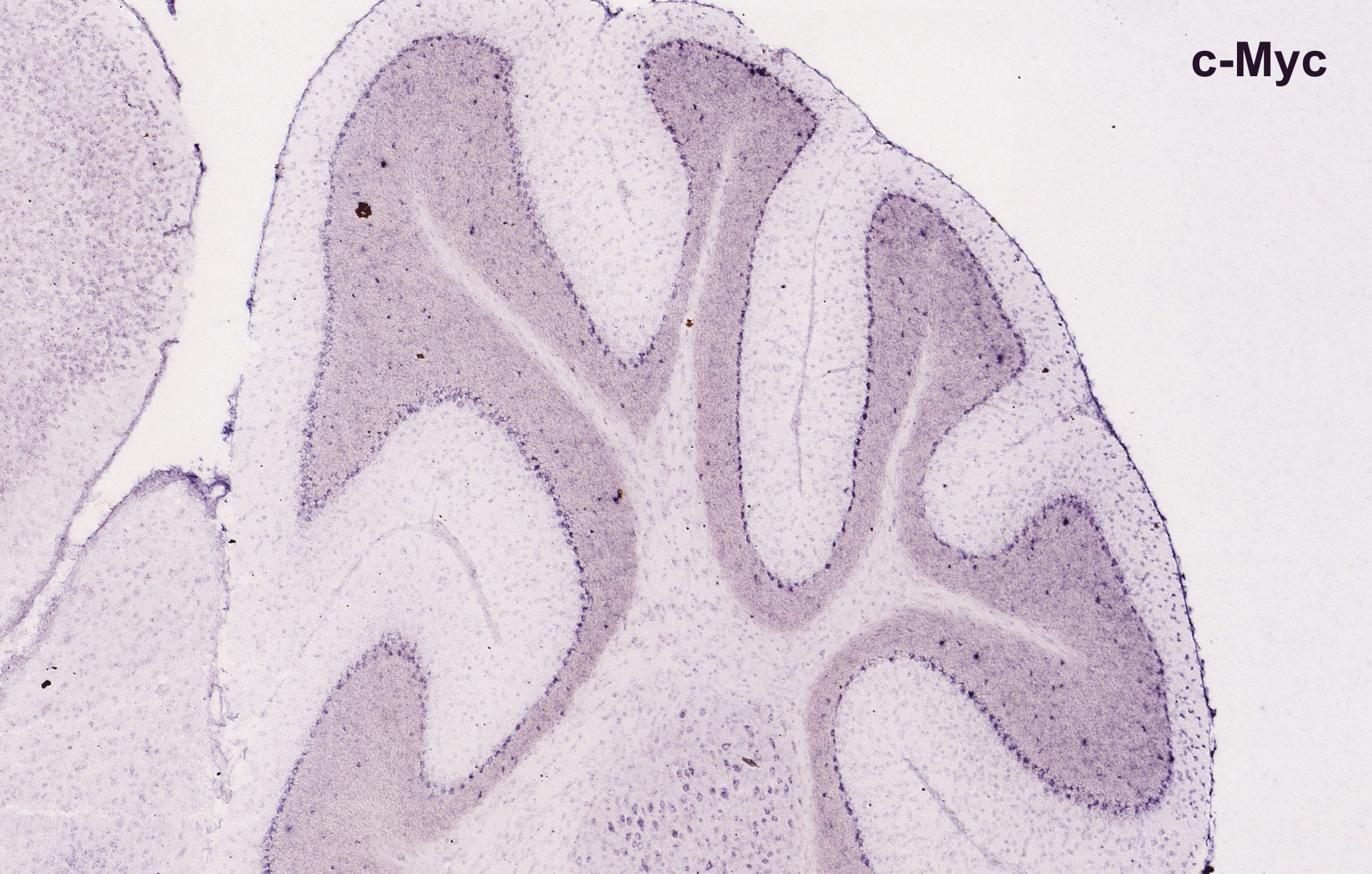

Supplement: Supplementary file 1 [file stem0027-1899-SD1.tif]

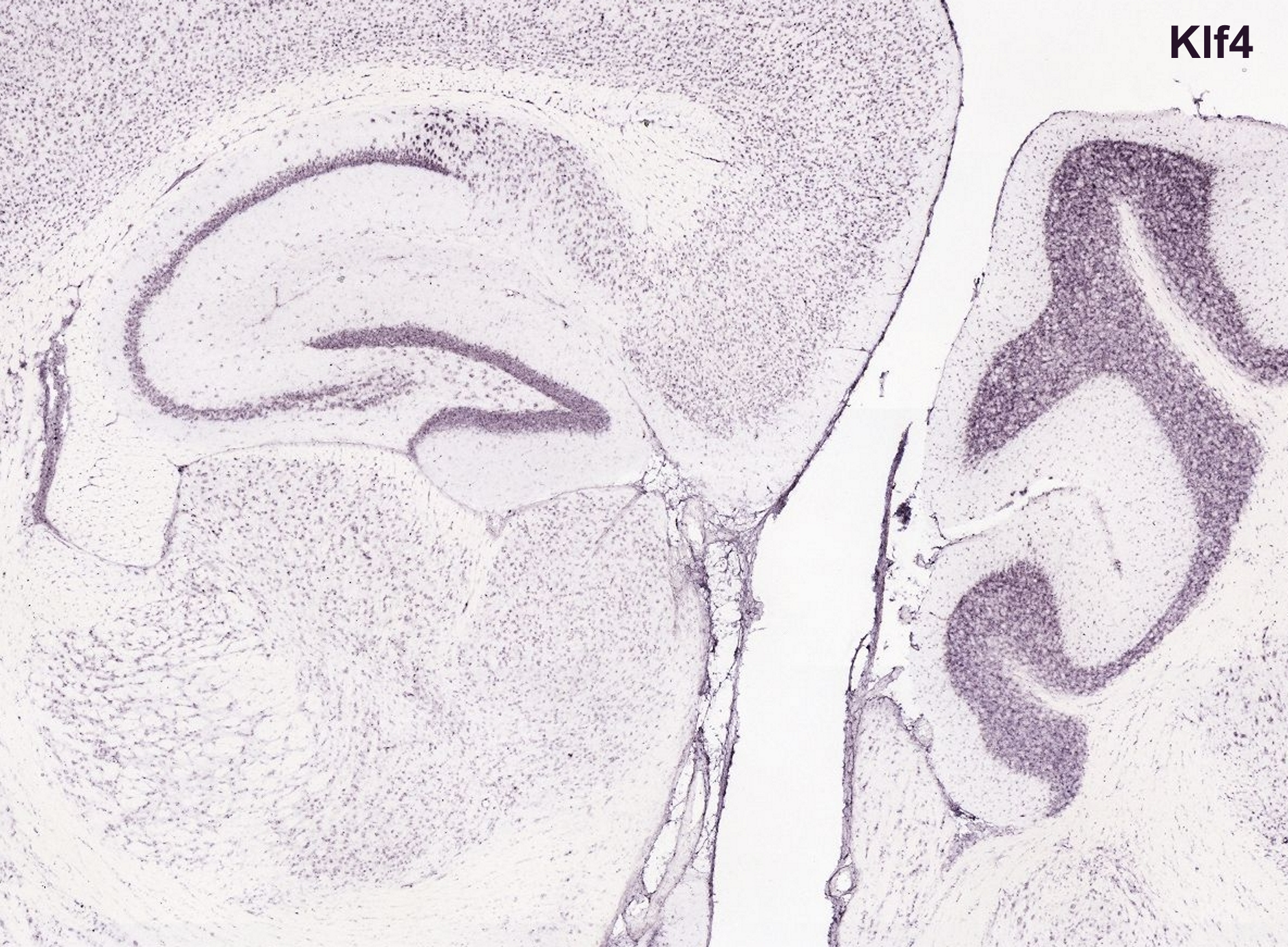

Supplement: Supplementary file 2 [file stem0027-1899-SD2.tif]

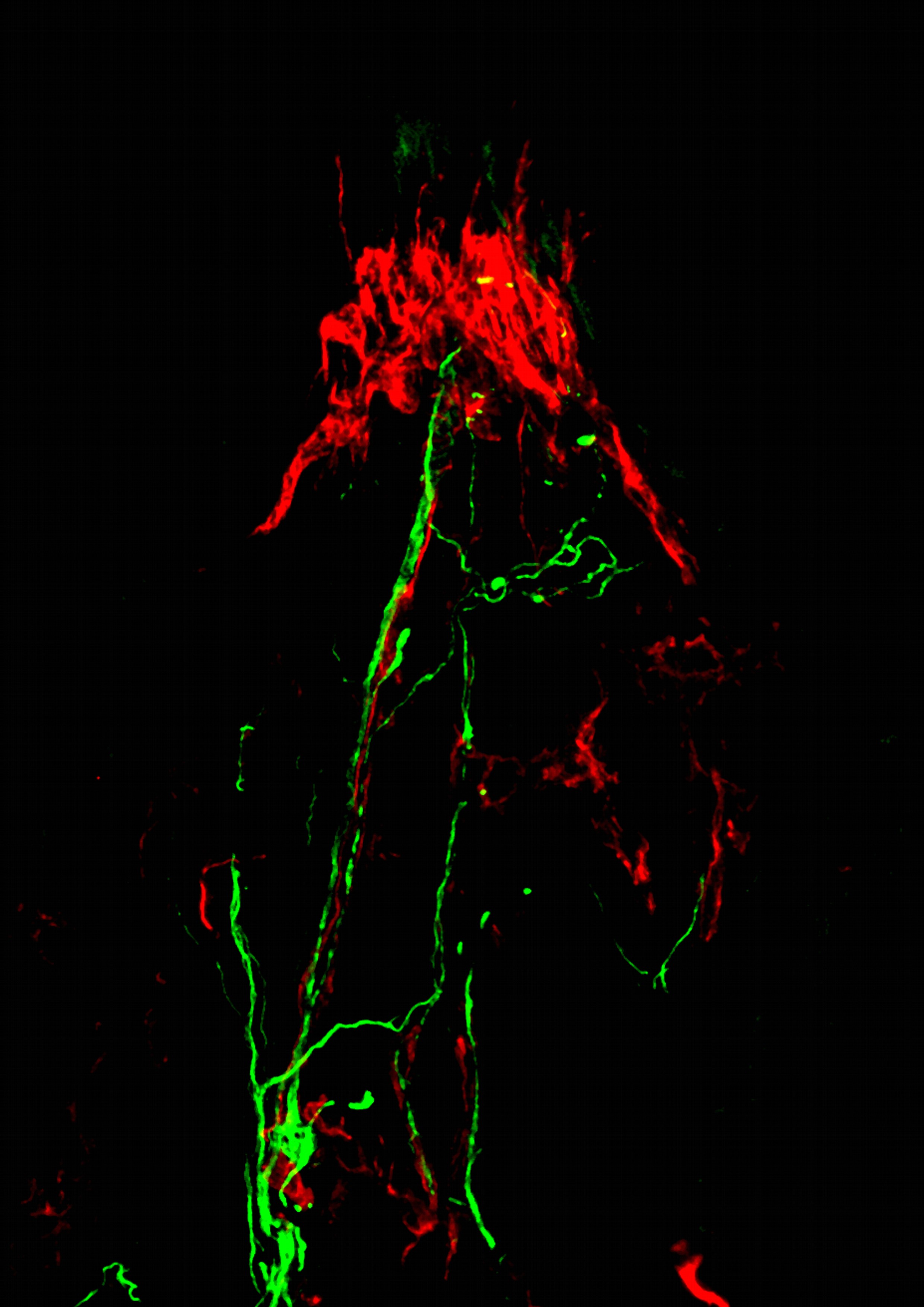

Supplement: Supplementary file 3 [file stem0027-1899-SD3.jpg]
