# Supplementary material for: Adult Palatum as a Novel Source of Neural Crest-Related Stem Cells
Source: Stem Cells. 2009 Aug;27(8):1899–910. doi: 10.1002/stem.104 (PMC2798069; doi:10.1002/stem.104)
Supplement: Supplementary file 4 [file stem0027-1899-SD4.doc]

| ***Name*** | ***sense primer*** | ***antisense primer*** |
| --- | --- | --- |
| ß-Actin | gag aag atg acc cag atc atg t | cat ctc ttg ctc gaa gtc cag |
| Twist | cac gct gcc ctc gga caa g | gtg ccc cac gcc ctg att ct |
| Sox2 | gca cat gaa cgg ctg gag caa cg | tgc tgc gag tag gac atg ctg tag g |
| Sox9 | gtg ctg aag ggc tac gac tgg a | gtt gtg cag atg cgg gta ctg g |
| ABCG2 | cca tag ccg cag gcc aaa gc | ggg cca cat gat tcc tcc ac |
| Oct4 | ggc gtt ctc ttt gca aag gtg ttc | ctc gaa cca cat cct tct ct |
| P75 | aca tac tca gac gaa gcc aac cac | cag ctg ttc cac ctc ttg aaa gca |
| Slug | caa cta cag cga act gga cac aca | aaa gcc ttg cca cag atc ttg c |
| Klf4 | aag ttt gtg ctg aag gcg tct ctg | aag tgt ggg tgg ctg ttc ttt tcc |
| c-myc | tgc gat cct gac gat gag acc tt | gtt gcc tct ttt cca cag aca cca |
| Nestin | cta cca gga gcg cgt ggc | tcc aca gcc agc tgg aac tt |
| Notch1 | caa tca ggg cac ctg tga gcc cac at | tag agc gct tga ttg ggt gct tgc gc |
| Notch2 | cat tga cga gtg cac tga | gag tgc tgg cac aag tgt |
| GFAP | gag atc gcc acc tac agg aa | gca cac ctc aca tca cat cc |
| ß-III-Tubulin | aac cag ata ggg gcc aag tt | ggc ctg aat agg tgt cca aa |
| hNestin | cag cgt tgg aac aga ggt tg | gct ggc aca ggt gtc tca ag |
| hSox2 | tgc agt aca act cca tga cca | gtg ctg gga cat gtg aag tct |
| CD133 | cac cgc tct aga tac tgc tgt tga | tga tgg acc atg gac tat aac gtg |
| human c-Myc | agg aga cat ggt gaa cca gag t | agc ctg cct ctt ttc cac aga aac |
| human Klf4 | tct cca att cgc tga ccc atc ct | ttc agc acg aac ttg ccc atc a |
| human Oct4 | ctc ctg aag cag aag agg atc ac | ctt ctg gcg ccg gtt aca gaa cca |
| human Actin | gag aag atg acc cag atc atg t | cat ctc ttg ctc gaa gtc cag |
|  |  |  |
|  |  |  |
|  |  |  |
|  |  |  |
